# Pharmacological mechanisms by which baicalin ameliorates cardiovascular disease

**DOI:** 10.3389/fphar.2024.1415971

**Published:** 2024-08-09

**Authors:** Lujia Si, Yu Lai

**Affiliations:** ^1^ Acupunture and Tuina School, Chengdu University of Traditional Chinese Medicine, Chengdu, China; ^2^ School of Basic Medicine, Chengdu University of Traditional Chinese Medicine, Chengdu, China

**Keywords:** baicalin, cardiovascular disease, cardiomyocyte, inflammation, oxidative stress

## Abstract

Baicalin is a flavonoid glycoside obtained from the dried root of *Scutellaria baicalensis* Georgi, which belongs to the Labiatae family. Accumulating evidence indicates that baicalin has favorable therapeutic effects on cardiovascular diseases. Previous studies have revealed the therapeutic effects of baicalin on atherosclerosis, myocardial ischemia/reperfusion injury, hypertension, and heart failure through anti-inflammatory, antioxidant, and lipid metabolism mechanisms. In recent years, some new ideas related to baicalin in ferroptosis, coagulation and fibrinolytic systems have been proposed, and new progress has been made in understanding the mechanism by which baicalin protects cardiomyocytes. However, many relevant underlying mechanisms remain unexplained, and much experimental data is lacking. Therefore, further research is needed to determine these mechanisms. In this review, we summarize the mechanisms of baicalin, which include its anti-inflammatory and antioxidant effects; inhibition of endothelial cell apoptosis; modulation of innate immunity; suppression of vascular smooth muscle cells proliferation, migration, and contraction; regulation of coagulation and fibrinolytic systems; inhibition of myocardial hypertrophy; prevention of myocardial fibrosis; and anti-apoptotic effects on cardiomyocytes.

## 1 Introduction

Cardiovascular diseases (CVDs) are defined as a variety of illnesses affecting the heart and blood vessels and include coronary heart disease, cerebrovascular disease, and rheumatic heart disease. Notably, CVDs cause the deaths of approximately 17.9 million individuals worldwide each year, rendering these diseases the most prevalent noncommunicable diseases and leading causes of mortality (Tsao et al., 2023). This finding indicates that CVDs impose significant health and financial costs. Currently, the use of Western medicine alone is no longer sufficient to control CVDs. The search for effective complementary and alternative therapies is warranted (Slostad et al., 2020). Chinese herbal medicine (CHM) has a rich history and has been a fundamental component of traditional Chinese healthcare for centuries. It has excellent results in the treatment of CVDs (Wal et al., 2024).

*Scutellaria baicalensis* Georgi, sometimes referred to as Huang-qin or Chinese skullcap, is obtained from the dehydrated root of *Scutellaria baicalensis*, which belongs to the Labiatae family. Baicalin is the main metabolite of *S. baicalensis* extract. Previous studies have summarized the significant clinical potential of baicalin in the treatment of atherosclerosis (AS), myocardial ischemia‒reperfusion injury (MIRI), hypertension, and heart failure through mechanisms including the regulation of lipid metabolism, the reduction of inflammation-induced damage, the inhibition of oxidative stress, the attenuation of apoptosis, and immune modulation (Xin et al., 2020). However, the specific signaling pathways involved have not been identified. In recent years, some new findings have been published on the effects of baicalin on coagulation and fibrinolytic mechanisms, as well as on its toxicity. Studies related to the anti-inflammatory, antioxidant, and immune system effects of baicalin have also been conducted. We summarize the mechanisms of baicalin, which include its anti-inflammatory and antioxidant effects; inhibition of endothelial cell apoptosis; modulation of innate immunity; inhibition of VSMC proliferation, migration, and contraction; modulation of coagulation and fibrinolytic systems; inhibition of myocardial hypertrophy; inhibition of myocardial fibrosis; and anti-apoptotic effects on cardiomyocytes, to synthesize the latest views and provide a theoretical basis for targeted therapy in animal experiments and clinical studies.

### 1.1 Methodology

In this study, information was obtained from PubMed and the Web of Science using “baicalin” as the search term. The most recent data collected were from studies published up to May 2024. This publication mentioned 136 items in total, of which 69 were research studies and five were reviews on baicalin. Of the 69 research, 37 focused on the mechanism of action of baicalin in preventing CVDs. Based on these studies, we clarified the research direction of the mechanisms related to the protection of the cardiovascular system by baicalin and defined the basic structure of this paper.

The inclusion criteria were that the study had to investigate the pharmacological effects of baicalin on cardiovascular diseases and the underlying mechanisms involved. Studies that did not include baicalin as a single active metabolite were excluded. The aim of this review is to facilitate the development of clinical applications of baicalin by fully analyzing the included literature and providing a useful reference for future studies (Table 1).

**TABLE 1:** Cardioprotective mechanisms of baicalin.

Pharmacological effects	Models/targets	Dosage	Molecular mechanisms	References
Antioxidant	C57BL/6 mice HUVECs HAOECs	50 mg kg^-1^ 50 μM	Reductions in ROS and proinflammatory factor production through Akt/GSK3B/Fyn-mediated Nrf2 activation	Chen et al. (2018)
	ApoE^−/−^ mice	50 mg kg^-1^	Inhibition of the NF-κB and p38 MAPK signaling pathways and upregulation of the activities of the antioxidant enzymes SOD, CAT and GSH-Px	Wu et al. (2018b)
	HUVECs	20 μM	Decreased NADPH oxidase activity and ROS production	Tsai et al. (2021)
	HAEC	0–400 μM	Increased NOX4 and eNOS production, decreased ROS production	Li and Ren (2022)
Anti-inflammatory	BMECs	10, 100 μM	Downregulated the MAPK and NF-κB signaling pathways, inhibited the nuclear translocation of NF-κB p65 and p-IκBα, impeded the phosphorylation of MRK1/2, ERK and p38	Zhang et al. (2013)
	RAW264.7 cells	0.1–1.0 μM^-1^	Inhibition of NF-κB p65 and IκB-α phosphorylation	Yan et al. (2021)
	LPS-induced HUVECs	5, 10 μM	Inhibited the ERK pathway and IL-6 and TNF-α expression	Kwak et al. (2014)
	ApoE^−/−^ mice	20, 50, 100 mg kg^-1^	Inhibited NLRP3 and reduced IL-1β, IL-18 and ROS production	Duewell et al. (2010)
	L-glutamate-induced HT-22 cells	4, 8, 16 μM	Inhibition of NLRP3 activation through the Nrf2/HO-1 pathway	Li et al. (2023)
	Raw264.7 cells	100 μM	Inhibited the PERK/TXNIP/NLRP3 Axis	Fu et al. (2021)
Inhibition of endothelial cell apoptosis	H/R-exposed HAECs	10, 20, 40 μM	Inhibition of PKCδ/p53 apoptosis signaling pathway activation	Shou et al. (2017)
	CMECs I/R rats	6.3, 25, 100 400 μg mL^-1^ 10, 30, 100 mg kg^-1^	Baicalin prevented necrotic apoptosis by inhibiting the RIP1, RIP3, and p-MLKL proteins	Bai et al. (2019)
	Thrombin-induced HUVECs	50 μM, 100 μM, 150 μM	Inhibition of NF-κB p65 phosphorylation and PAR-1 expression	Zhang et al. (2019)
Effects on macrophages	Human monocyte THP-1 cells	0–200 μM	Modulating the PPAR-γ/LXRα/ABCA1/ABCG1 pathway enhanced cholesterol efflux	He et al. (2016)
	THP-1 macrophages	2, 10, 50 µM	THP-1 macrophage induction of cholesterol efflux through the PPAR-γ/LXRα/SR-BI pathway	Yu et al. (2016)
	MØ-MP- induced RAW264.7 cells	1, 2.5, 5, 10, 25 µM	Suppressed the production of NO, foam cells, apoptosis, ROS, metalloproteinase-9 expression, and VSMC proliferation	Paudel and Kim (2020)
	BM-MNC cells	50 μM	Upregulated MERTK, PTX3, and IL-10	Lai et al. (2018)
	I/R rats	20, 60, 120 mg kg^-1^	Decreased the levels of cleaved capase3, iNOS, and IL-1β and increased the levels of Arg-1 and IL-10	Xu et al. (2020)
Inhibition of VSMC proliferation, migration, and hypercontraction	PDGF-BB induced VSMC	1–60 μM	Inhibited PDGFRβ-ERK signaling and increased p27 levels	Dong et al. (2010)
	Sm22α−/− mice	70 mg kg^-1^	Inhibition of VSMC proliferation and migration by upregulating SM22α expression and blocking the Ras-Arp2/3 signaling pathway	Lv et al. (2016)
	HUVECs	6.25, 12.5, 25, 50 mmol L^-1^	The expression of Bax, Bcl-2, and caspase-3 was downregulated; the ACE2/Ang-(1–7)/Mas axis was activated; and the PI3K/AKT/eNOS pathway was upregulated	Wei et al. (2015)
	Ang II-induced C57BL/6 mice Ang II-induced VSMCs	*In vivo*: 5 mg kg^-1^ *In vitro*: 12.5, 25, 50 μM	Attenuated Ang II-induced intracellular Ca2+ release, AT1R expression, and the activation of the MLCK/p-MLC signaling pathway	Liu et al. (2021)
	SHR rats	50, 100, 150 mg kg^-1^	Regulated Ang II, activated KATP	Ding et al. (2019)
Regulation of coagulation and fibrinolytic systems	HUVECs	0–50 μM	Inhibited PAI-1 expression, inhibited thrombin and FXa activity, and reduced the PAI-1/t-PA ratio	Lee et al. (2015b)
	LPS-induced rats TNF-α-induced HUVECs	*In vivo*:50, 100 mg kg^-1^ *In vitro*:10, 20, 50 μM	Suppressed the AKT/Ca2+/ROS pathway in platelets and the Furin/TGFβ1/Smad3/TSP-1 pathway in ECs	Wang et al. (2023a)
	OA-induced ARDS rats	150, 300, 450 mg kg^-1^	Inhibition of MPO activity	Zhao et al. (2017)
Inhibition of cardiac hypertrophy	C57BL/6J mice	100 mg kg^-1^	Induced PPARα and PPARβ/δ expression	Zhang et al. (2017)
	Hypoxia-induced PH rats	10, 20, 30 mg kg^-1^	Inhibition of the HMGB1/RAGE signaling pathway to block PPARγ activation	Chen and Wang (2017)
Inhibition of myocardial fibrosis	Renovascular hypertensive rats	100 mg kg^-1^	Inhibited MMP-9, MMP-2, CTGF and TGF-β1 expression	Dai et al. (2017)
	Hypoxia-induced pulmonary hypertension in rats	30 mg kg^-1^	Downregulated the p38 MAPK/MMP-9 pathway	Yan et al. (2016)
Inhibition of cardiomyocyte apoptosis	Myocardial infarcted rats	50, 100, 200 mg kg^-1^	Increased the ERK phosphorylation level and inhibited p-JUNK and p-p38 expression	Liu et al. (2013)
	Sprague–Dawley rats I/R cells	*In vivo*: 100 mg kg^-1^ *In vitro*: 10 μM	Inhibited the CaSR/ERK1/2 signaling pathway	Liu et al. (2020)
	H9c2 cardiomyocytes	10 μM	Inhibition of STIM1 reduced the intracellular calcium concentration	Li et al. (2019b)
	H9c2 cardiomyocytes	10 μM	Suppression of NF-κB nuclear translocation	Lin et al. (2010)
	DOX-induced cardiotoxicity in rats	100 mg kg^-1^	Inhibition of the TLR4/IκBα/NFκB signaling pathway	Feng et al. (2022)
	H9c2 cardiomyocytes	25, 50, 75, 100 μM	Activates the Nrf2/HO-1 pathway and upregulates HIF1α	Yu et al. (2019)
	Post-CA rats	100 mg kg^-1^	Drp1-mediated mitochondrial fission inhibition circumvented ischemic myocardial damage following CA	Wu et al. (2021)
	H/R-induced H9c2 cardiomyocytes	10, 20 μM	Enhanced ALDH activity in H9c2 cardiomyocytes	Jiang et al. (2018)
	myocardial I/R rats OGD/R H9c2 cells	100, 200 mg kg^-1^	Inhibition of TfR1 signaling activation and ferritin-mediated autophagy through NCOA4	Fan et al. (2021)

### 1.2 Chemical properties of baicalin

**FIGURE 1:**
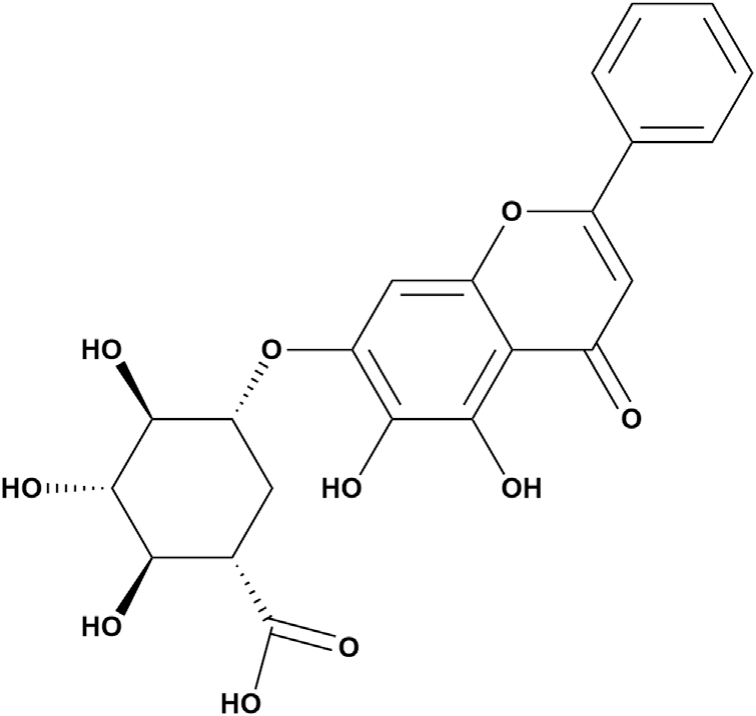
Chemical structure of baicalin.

Baicalin is a flavonoid glycoside obtained from the dried root of *S. baicalensis*, which belongs to the Labiatae family (Wang et al., 2018) (Figure 1). It is created through the fusion of baicalein with a glucuronic acid molecule; multiple phenolic hydroxyl groups are contained within the molecule, and the structure is an essential basis for its antioxidant function. In addition, the hydroxyl group and the 8′and 4′positions on the baicalin A ring are the main active metabolic sites of baicalin. β-Glucuronidase, UGT, sulfatase, and catechol-O-methyltransferases are also involved in this active metabolic process (Lu et al., 2012; Wang et al., 2012; Akao et al., 2013). Its concentration ranges from 8.1% to 15.6% (Sowndhararajan et al., 2018). Notably, the amount of baicalin extracted from *S. baicalensis* exhibits significant annual variation. According to quantitative HPLC studies, the amount of baicalin in *S. baicalensis* increases consistently from the first year to the second year of cultivation and decreases from the third year to the fourth year (Zhang N. et al., 2022).

Baicalin is a yellow crystalline compound with a molecular weight of 446.4 g/mol. It features a melting point range of 202°C–205°C and can readily dissolve in alkaline solutions (Ganguly et al., 2022). The physicochemical properties of baicalin were determined in 0.1 M NaCl at 25°C, and were pKa1 = 7.6, and pKa2 = 10.1 (Liang et al., 2009). However, it has low water solubility (67.03 ± 1.60 μg/mL) and low permeability (*P*_app_ = 0.037 × 10^−6^ cm/m) (Wu et al., 2014). Consequently, the oral administration of baicalin results in limited absorption. Following the initial glucuronidation, the bioavailability of baicalin was only 2.2% (Xing et al., 2005). Recent studies have shown that the water solubility of baicalin can be enhanced by glucosylation. Researchers have synthesized two glucosides, baicalein-7-O-α-D-glucuronidyl-(1→4′)-O-α-D-glucopyranoside (BG1) and baicalein-7-O-α-D-glucuronidyl-(1→4′)-O-α-D-maltoside (BG2). After dissolving them in water, transglucosylated BG1 was 188 times more soluble than baicalin, and BG2 was 320 times more soluble than baicalin. After antioxidant and antiglycation evaluations, BG1 and BG2 were found to have the same antioxidant and antiglycation properties as baicalin (Lambert et al., 2023). Considering the physicochemical properties of baicalin and the needs of clinical applications, studies of related drug delivery systems are necessary are urgently needed.

### 1.3 Drug delivery systems for baicalin

Nanosizing technology can facilitate the dissolution of difficult-to-solve drugs and effectively improve bioavailability. Researchers have used liposomes, solid nanoparticles, nanocrystals, and nanosolutions as drug delivery systems based on the properties of baicalin. Liposomes are the most widely studied nanodrug delivery system and have excellent biocompatibility, hydrophilicity, and low toxicity. Compared with a carboxymethyl cellulose suspension containing BA (BA-CMC), the oral bioavailability and peak concentration (C_max_) of BA-loaded liposomes (BA-LPs) were three and 2.82 times higher than those of BA-CMC, respectively. The *in vivo* distribution results showed that the concentrations of BA-LPs in the liver, kidney, and lung were 5.59, 2.33, and 1.25 times higher than those of BA-CMC, respectively (Wei et al., 2014). Liposomes not only improve drug utilization but also have targeting properties. The injection of BA-LPs increased the concentrations of baicalin in the brain, liver, spleen, heart, and lung in both the normal and MCAO models, but it lowered the concentrations in the kidney. The area under the concentration–time curve (AUC_(0-t)_) suggested that BA-LPs help to prolong the retention time of the drug in the body (Li et al., 2018). Solid lipid nanoparticles (SLNs) are colloidal lipid nanocarriers for pharmaceutical use. They are made of solid lipids with high biocompatibility. Zhidong Liu et al. used emulsion–evaporation–solidification at low temperatures to prepare PEGylated cationic solid lipid nanoparticles loaded with baicalin (OX26-PEG-CSLN). The pharmacokinetic results showed that the AUC_(0-t)_ of OX26-PEG-CSLN was 11.08-fold higher than that of baicalin solution (SOL), and the Cmax of OX26-PEG-CSLN was 7.88-fold higher than that of SOL (Liu et al., 2015). With a particle size of only 100 nm, baicalin-loaded PEGylated nanostructured lipid carriers (BN-PEG-NLCs) are able to cross blood vessels and evade macrophages. Compared to baicalin, BN-PEG-NLCs showed a 2.9-fold increase in drug concentration in the heart. The highest concentration of BN-PEG-NLCs was detected in the heart, demonstrating good targeting of the heart (Zhang et al., 2016). Nanoemulsions have good solubility and stability and can improve the therapeutic efficacy of drugs. After oral administration of 100 mg/kg baicalin-loaded nanoemulsion to rats, the plasma drug concentration–time profiles were 7 times higher than those of the baicalin suspension, and the *in vitro* release results showed a slow release property (Zhao et al., 2013). Lei Wu et al. used the *in situ* single-pass intestine perfusion (SPIP) method and a chylomicron-blocked rat model to study the properties of a baicalin nanoemulsion. The pharmacokinetic study showed that compared with a baicalin suspension, a baicalin nanoemulsion showed a 3.39-fold increase in the plasma C_max_, a 3-fold increase in T_max_, and a 14.56-fold increase in AUC_(0-t)_ (Wu L. et al., 2018). Although a wide variety of baicalin-loaded nanopreparations have been shown to significantly enhance the bioavailability of baicalin, these experiments did not compare the effects of different nanopreparations. The characteristics of different nanoformulations and the variability they exhibit *in vivo* experiments are issues that need to be explored and investigated in depth.

In addition, other drug delivery systems have been effective at improving the bioavailability of baicalin. The microsponge prepared by liquid‒liquid suspension polymerization and the quasiemulsion solvent diffusion method has porous channels. The slow release effect of baicalin could be enhanced by encapsulating baicalin, resulting in an average cumulative release rate of 85.92% ± 0.72% of baicalin over 36 h. The microsponge was also used to enhance the bioavailability of baicalin by encapsulating baicalin. An *in vitro* anti-inflammatory assay showed that the baicalin microsponge did not affect the anti-inflammatory effect of baicalin (Li M. et al., 2024). Cyclodextrins (CDs) are cyclic oligosaccharides composed of α-1,4-linked D-glucopyranose. CDs can improve the permeation and water solubility of baicalin. Compared with baicalin, the solubility of the baicalin-γ-CD complex increased 5.47-fold, and the baicalin-γ-CD complex had better stability (Jakab et al., 2019). We have summarized the drug delivery systems for baicalin. The results of the respective studies suggest a significant increase in the bioavailability of baicalin in the presence of the drug delivery system (Table 2). However, these delivery systems did not illustrate the variability of the concentrations distributed to various organs in the experiments, and further refinement is needed.

**TABLE 2:** Summary of baicalin nanoformulations.

Nanoformulations	Method of preparation	Outcomes	References
Liposomes	Effervescent dispersion technique	The oral bioavailability and Cmax of BA-LPs were 3-fold and 2.82-fold higher than those of BA-CMC, and the drug concentrations in the liver, kidney, and lung were 5.59-fold, 2.33-fold, and 1.25-fold higher than those of BA-CMC, respectively	Wei et al. (2014)
	Reverse evaporation	The Cmax and AUC(0-t) values of BA-LPs were 37.21 μg/mL and 2,266.38 min-µg/mL, respectively, which were significantly higher than the baicalin solution	Li et al. (2018)
Solid lipid nanoparticles	Emulsion, evaporation, and solidification at a low temperature	The AUC(0-t) and Cmax of OX26-PEG-SNLs were 5.69-fold and 6.84-fold higher than those of the baicalin solution, respectively	Liu et al. (2015)
	Emulsion-evaporation and low-temperature solidification method	BN-PEG-NLCs were targeted at the heart, and the highest concentration was distributed in the heart. Compared to a baicalin solution, BN-PEG-NLCs showed a 2.9-fold increase in the drug concentration in the heart	Zhang et al. (2016)
Nanoemulsions	Pseudoternary phase diagrams	Blood concentrations of baicalin-loaded nanoemulsions, including BAN-1 and BAN-2, reached 3.155 ± 0.132 mg/L and 4.625 ± 0.203 mg/L, which were 3–4 times higher than those of baicalin	Zhao et al. (2013)
	Pseudoternary phase diagrams	Compared to a baicalin suspension, baicalin nanoemulsions showed a 3.39-fold increase in the plasma Cmax, a 3-fold increase in Tmax, and a 14.56-fold increase in AUC (0-t)	Wu et al. (2018a)

### 1.4 Biopharmaceutics of baicalin

Baicalin cannot be effectively absorbed through the digestive system. The strong polarity of this substance prevents it from diffusing passively across the lipid bilayer. Although baicalin is absorbed in the stomach, this process is limited to the small and large intestines (Ganguly et al., 2022). Organs or tissues cannot absorb baicalin directly from the intestines. Instead, the intestinal tract transforms it into baicalein for use in the case of baicalin malabsorption. Due to its lipophilicity, the glycoside of baicalin, known as baicalein, exhibits excellent permeability and is more efficiently absorbed by the gastrointestinal system (Noh et al., 2016). Upon comparing the absorption of baicalein and baicalin in the gastrointestinal system, baicalein outperformed baicalin in every section of the gut (Cai et al., 2016).

**FIGURE 2:**
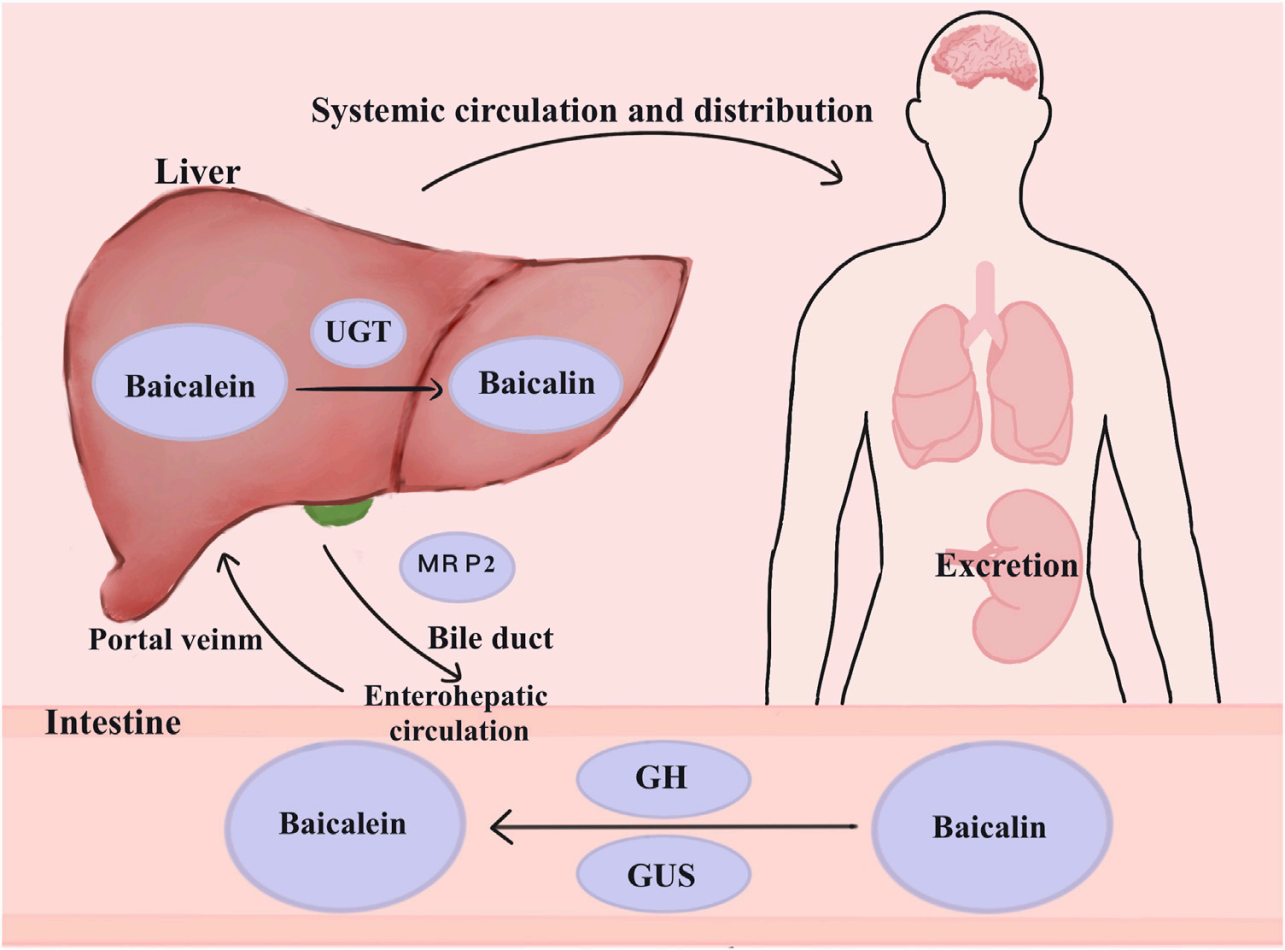
Metabolism of baicalin in the body. Baicalin is changed into baicalein in the presence of GH and GUS in the colon, where it then enters the enterohepatic circulation. UGT causes the conversion of glucuronidation to baicalin. By affecting bile excretion, MRP2 controls the enterohepatic circulation’s baicalin levels. Baicalin can also be eliminated via the kidneys. Abbreviations: multidrug resistance-associated proteins 2 (MRP2), β-glucuronidase (GUS), UDP-glucuronosyltransferase (UGT), glycoside hydrolase (GH).

The primary metabolic pathway for baicalin is glucuronidation. Oral baicalin is transformed to baicalein first by the action of β-glucuronidase (GUS) and glycoside hydrolase (GH), which are produced by the gut flora. Baicalein is absorbed in the intestine and reconverted to baicalin-7-glucuronide (baicalin), baicalein 6-O-glucuronide, and trace amounts of baicalein-6,7-diglucuronide by the action of UDP glucuronosyltransferase (UGT) in the small intestine or liver (Kang et al., 2014). Once glucuronidated, baicalin is transferred by the enterohepatic circulation, which involves the liver taking up the drug from the blood, processing it, and excreting bile. The multidrug resistance protein 2 (MRP2) transporter protein influences the process of biliary excretion (Akao et al., 2009). Then, the duodenum reabsorbs the drug, and the liver takes it up via the portal vein. Additionally, baicalin can be excreted through the urine (Figure 2).

## 2 Mechanism of action of baicalin on blood vessels

**FIGURE 3:**
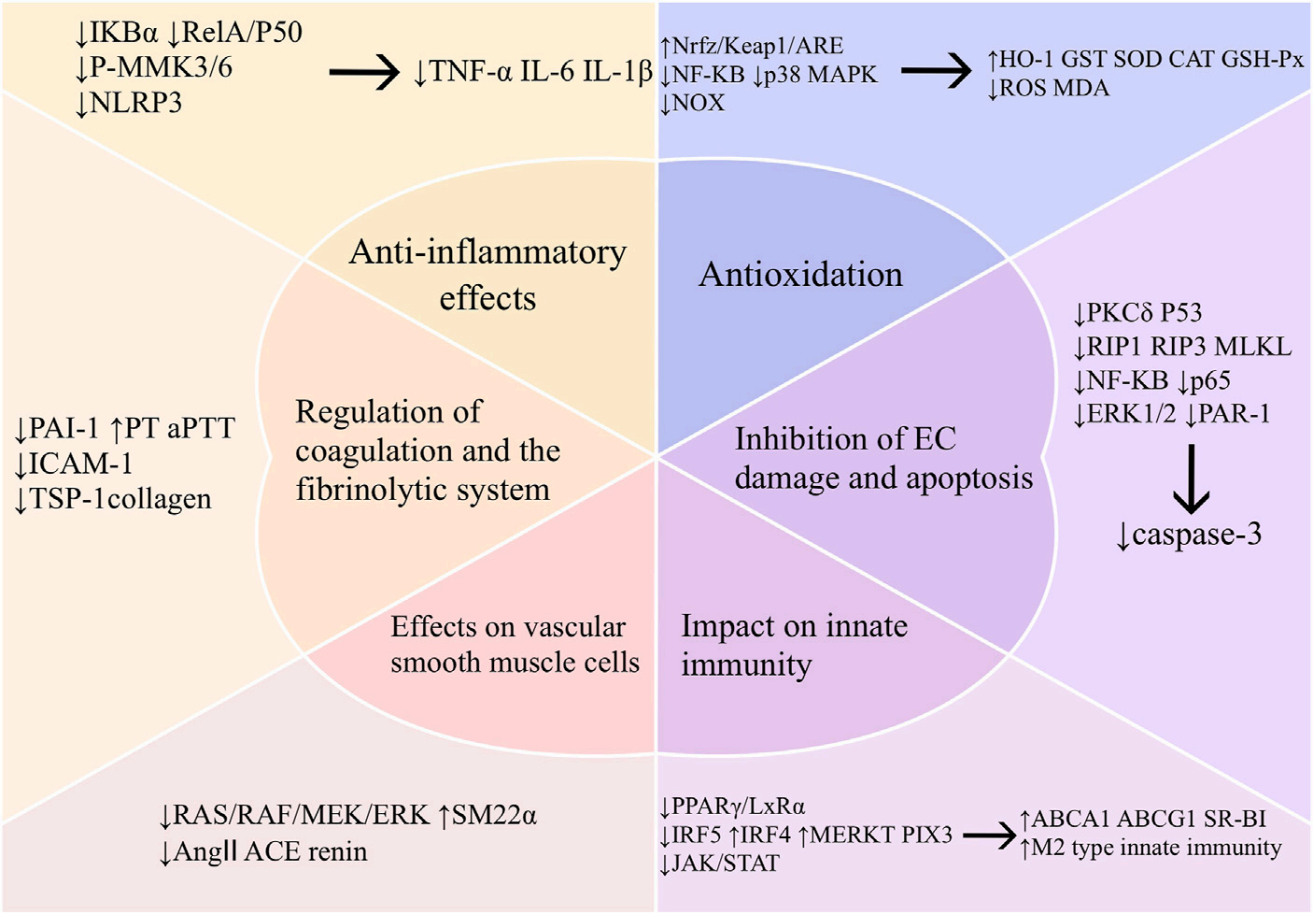
Mechanism of vascular protection by baicalin. Abbreviations: TNF-α (tumor necrosis factor-α), IL-6 (interleukin-6), IL-1β (interleukin-1β),mitogen-activated protein kinase kinase 3 (MKK3), mitogen-activated protein kinase kinase 6 (MKK6), nucleotide-binding oligomerization domain-like receptor protein 3 (NLRP3), oxidative stress (OS), malondialdehyde (MDA), superoxide dismutase (SOD), glutathione peroxidase (GSH-px), heme oxygenase-1 (HO-1), glutathione S-transferase (GST), CAT (Catalase), NADPH oxidase (NOX), protein kinase C-delta (PKCδ), receptor-interacting protein 1 (RIP1), receptor-interacting protein 3 (RIP3), mixed lineage kinase domain-like protein (MLKL), extracellular regulated protein kinases (ERK), protease activated receptor-1 (PAR-1), ATP binding cassette subfamily A member 1 (ABCA1), ATP binding cassette subfamily G member 1 (ABCG1), scavenger receptor class B type I (SR-BI), peroxisome proliferator-activated receptor-γ (PPAR-γ), liver X receptor α (LXRα), interferon regulatory factor 5 (IRF5), interferon regulatory factor 4 (IRF4), myeloid-epithelial-reproductive tyrosine kinase (MERTK), pentraxins 3 (PTX3), janus kinase 2 (JAK2), signal transducer and activator of transcription 3 (STAT3), smooth muscle 22α protein (SM22α), mitogen-activated proteinkinase kinase 1 (MEK), angiotensin I-converting enzyme (ACE), angiotensin II (Ang II), plasminogen activator inhibitor-1 (PAI-1), prothrombin time (PT), activated partial thromboplastin time (aPTT), intercellular cell adhesion molecule-1 (ICAM-1), thrombospondin-1 (TSP-1).

Inflammation and oxidative stress play important roles in the development of intravascular injury. Vascular endothelial cells, vascular smooth muscle cells, macrophages, and coagulation and fibrinolytic systems are also closely related to vascular health. In recent years, numerous studies have been conducted to elucidate the relevant mechanisms involved. We have provided a detailed description below for a better understanding of the mechanisms by which baicalin prevents vascular damage (Figure 3).

### 2.1 Anti-inflammatory effects

Inflammation plays an integral role in the onset and development of CVDs. Oxidative stress induced by low-grade inflammation is one of the causative factors of most CVDs (Steven et al., 2019). Studies have shown that the curative impact of baicalin on CVD treatment is associated with its detrimental effect on inflammation. In many of the animal models studied, baicalin considerably decreased the levels of the proinflammatory cytokines IL-1β, IL-6, and TNF-α. This effect modulated various signaling pathways associated with inflammatory responses. We have summarized the major relevant pathways below.

#### 2.1.1 NF-κB

The NF-κB-mediated signaling cascade is an important pathway that promotes the cardiovascular inflammatory response. Cytokines, including TNF-α, IL-1, and ox-LDL, together with reactive oxygen species (ROS), function as activators of NF-κB. NF-κB activity triggers the generation of inflammatory cytokines, chemokines, and adhesion molecules by immune cells, which include macrophages, neutrophils, and dendritic cells (Cheng et al., 2023). In the inactive state, NF-κB interacts with members of the IκB family to create a complex. Proinflammatory factors phosphorylate IKK kinase, promoting the degradation and phosphorylation of the IκB family. After formation, the NF-κB p65-p50 dimer migrates to the nucleus, where it interacts with specific genes and regulates gene expression (Zhang et al., 2013). These inflammatory responses ultimately lead to cardiovascular damage.

Inhibition of IκBα phosphorylation and the nuclear translocation of NF-κB are both effective means by which baicalin modulates the NF-κB pathway. According to *in vitro* experiments, 1.0 μmol/L baicalin effectively inhibited the phosphorylation of NF-κB p65 and IκBα and the expression of the inflammatory factors TNF-α, IL-1β, IL-6, Cox, and iNOS in RAW264.7 cells (Yan et al., 2021). The addition of 10 μM baicalin to rat brain microvascular endothelial cells (BMECs) significantly inhibited p-IKKα, p-IKKβ, and p-IκBα in the NF-кB signaling pathway and attenuated cellular inflammatory injury (Zhang et al., 2013). In the experiments performed by Peng Zhang et al., baicalin reduced the expression of p-IκBα in the nucleus by reversing the translocation of NF-κB p65 from the nucleus to the cytoplasm. This process resulted in a reduction in the levels of proinflammatory cytokines such as TNF-α, IL-1, and IL-6. Hypoxia- and glucose deficiency-induced damage to cerebral microvascular endothelial cells in rats were effectively attenuated (Zhang et al., 2013). In another experiment designed to study the anti-inflammatory effects of baicalin, HUVECs were treated with LPS (100 ng/mL) for 4 h, and then 10 μM baicalin was added and incubated for 6 h. The results showed that the baicalin treatment significantly inhibited the nuclear translocation of NF-κB compared with the HUVECs treated without baicalin. Baicalin also significantly reduced TNF-α levels compared with those in the control group treated with baicalein and wogonin (Lee et al., 2015a). Researchers have noted that the inhibition of NF-κB p65 nuclear translocation, as well as TNF-α and IL-6 expression, may be one of the mechanisms by which baicalin attenuates vascular inflammation.

Baicalin can additionally exert anti-inflammatory effects by suppressing the expression of target genes. Among them, the cytokines IL-1, IL-2, IL-6, and TNF-α, as well as the adhesion molecules intercellular adhesion molecule (ICAM), vascular cell adhesion molecule (VCAM), and E-selectin, are target genes regulated by NF-κB. Research on the relationship between baicalin and the lipopolysaccharide (LPS)-mediated vascular inflammatory response has shown that baicalin can directly inhibit the production of IL-6 and TNF-α. Furthermore, it reduces the expression of surface proteins such as VCAM-1, ICAM-1, and E-selectin in a concentration-dependent manner (Lee et al., 2015a). These multifaceted anti-inflammatory mechanisms indicate the therapeutic potential of baicalin for vascular inflammation.

#### 2.1.2 MAPK

The mitogen-activated protein kinase (MAPK) pathway, which can inhibit inflammation, is another component of the mechanism of action of baicalin. The MAPK pathway is a major intracellular signal transduction system that transmits signals from extracellular stimuli to the nucleus through a signaling cascade. Extracellular signal-regulated kinases (ERKs), including ERK1/2, p38, and c-Jun N-terminal kinases (JNKs), are the three primary members of this family of intracellular signaling molecules (Gaestel, 2015). Among these pathways, the p38 MAPK signaling pathway plays an essential role in modulating the inflammatory response. Proinflammatory cytokines activate MEKK1/4, and the phosphorylation of MEKK1/4 prompts the phosphorylation of MKK3/6. The phosphorylation of MKK3/6 initiates the translocation of p38 to the nucleus. In the nucleus, p38 then phosphorylates multiple transcription factors (Li X. et al., 2024). Research has indicated that the MAPK pathway is intricately linked to the development of inflammatory CVDs. In a rabbit model of AS, activation of the MAPK cascade caused an increase in the serum levels of inflammatory mediators, including IL-1β, IL-6, and TNF-α. These mediators promote the formation and exacerbation of lipid plaques (Song et al., 2013). Polyphenolic tannic acid inhibits TLR4 generation, reduces JNK phosphorylation, and decreases p38 protein production in mice with AS. Polyphenolic tannic acid alleviates macrophage inflammation related to AS by blocking the MAPK pathway (Meng et al., 2023). These findings suggest that inhibiting the MAPK pathway to induce inflammation is effective for suppressing the CVD incidence.

The signaling cascade of the MAPK pathway is closely related to phosphorylation. Reducing inflammation requires the inhibition of MAPK pathway-mediated phosphorylation. This pathway is centered on p38, which is an essential effector. JNK and NF-κB can be activated when p38 is activated, leading to a series of inflammatory reactions. A study on the mechanisms of inflammation associated with AS also showed that 100 mg/kg baicalin significantly reduced p38 phosphorylation levels and inhibited the p38 MAPK signaling cascade to reduced atherosclerotic plaque progression in ApoE^−/−^ mice fed a high-fat diet (Wu Y. et al., 2018). In another modeling experiment, baicalin protected the vascular barrier by reducing the activity of phosphorylated p38 through the modulation of the p38 MAPK pathway both *in vitro* and *in vivo* (Kwak et al., 2014). When 10 μM and 100 μM baicalin were added to rat BMECs for 6 h, the researchers observed very interesting phenomena. Baicalin (100 μM) significantly downregulated the phosphorylation of proteins in the MAPK signaling pathway, such as p-MRK1/2, p-ERK, and p-p38. At 10 μM, baicalin better inhibited the NF-кB signaling pathway (Zhang et al., 2013). Although the use of different concentrations affected different signaling pathways, baicalin generally has a strong anti-inflammatory effect and has a good protective effect on OGD-injured BMECs.

#### 2.1.3 NLRP3

The nucleotide-binding oligomerization domain-like receptor protein 3 (NLRP3) inflammasome is an intricate protein assembly formed through the involvement of intracellular pattern recognition receptors (PRRs). These complexes play a crucial role in the immune system. ROS, cholesterol crystals (CCs), and low-density lipoprotein (LDL) are integral in the development of CVDs because they trigger the activation of the NLRP3 inflammasome (Xu et al., 2024). The activation process comprises two primary stages. Initially, TLR signaling is stimulated by antigenic substances, which then activate NF-κB. This mechanism produces precursors of IL-1β and IL-18, as well as an inactive variation of NLRP3. Afterward, upon exposure to antigenic substances, immune cells begin the process of oligomerizing the NLRP3 protein, which interacts with intracellular ASC and procaspase-1 to create the inflammasome. Throughout this process, many inflammatory cytokines, including IL-1β and IL-18, are produced and released (Shao et al., 2023). The IL-1 family of cytokines is directly related to the formation of atherosclerotic plaques. A significant reduction in the expression of various mRNAs, including caspase-1, ICAM, and VCAM, in NLRP3 knockdown mice is linked to the progression of AS (Zhao et al., 2020). After silencing NLRP3, the levels of IL-1β and IL-18 decreased, and ROS production was also blocked. An average 69% reduction in the atherosclerotic lesion area was observed in mice lacking the NLRP3 inflammasome or IL-1 compared with those from LDLR-deficient mice (Duewell et al., 2010). These studies indicate that NLRP3 inflammasome is a critical link in the atherosclerotic process.

Additionally, the NLRP3 inflammasome is strongly linked to pyroptosis. Pyroptosis is a mode of programmed cell death mediated by gasdermin (GSDM) (Yang and Jiang, 2024). The onset of pyroptosis prompts cell membrane rupture to release proinflammatory cytokines that induce and amplify inflammation. Pyroptosis encompasses a pair of main pathways: the conventional inflammatory vesicle pathway facilitated by caspase-1 and the noncanonical inflammatory vesicle pathway facilitated by caspase-4/5. In the classic pathway, macrophages, endothelial cells, and vascular smooth muscle cells undergo pyroptosis, which significantly stimulates the development of CVDs (Qiu et al., 2024). Pyroptosis is a pathological response of macrophages that phagocytose ox-LDL or CCs. It occurs when ox-LDL or CCs phagocytosed by macrophages cannot be digested. Massive pyroptosis destroys the number and structure of normal cells, releasing inflammatory mediators to exacerbate the inflammatory response. Research has shown that elevated intracellular ROS levels result in the death of macrophages. Elevated ROS levels trigger the activation of the NLRP3 inflammasome, the cleavage of caspase-1, and an increase in the synthesis of IL-1β, IL-18, and GSDMD (Mao et al., 2021). Ox-LDL activates the pyroptosis pathway controlled by NLRP3 in macrophages, culminating in increased instability of atherosclerotic plaques (Lin et al., 2021). Macrophage death was significantly inhibited when the expression of the inflammatory mediators NLRP3, caspase-1, and GSDMD was downregulated (Zhang S. et al., 2023). EC pyroptosis triggers an inflammatory response that disrupts EC integrity. The classic NLRP3-mediated pyroptosis pathway is the major pyroptosis pathway in ECs. Furthermore, G protein-coupled receptor 124 (GPCR124) promotes NLRP3, GSDMD, and caspase-1 activity during brain vascular cell injury caused by hypoxia or ischemia (Xu et al., 2023). These findings indicate that EC pyroptosis resulting in CVDs is closely related to the classic NLRP3 pathway.

Baicalin inhibits NLRP3 to fight inflammation in two ways, one of which involves directly reducing the production of inflammatory factors. The other is to control the damage caused by inflammation in the cardiovascular system. The inhibitory effect of baicalin on the NLRP3 inflammasome can effectively reduce the damage caused by the release of inflammatory factors from inflammatory vesicles. After the administration of 100 mg/kg baicalin, IL-1β and IL-18 levels were significantly reduced in AS mice. NLRP3 protein and mRNA levels appeared to be decreased, and the fluorescence signals of ROS and total ROS in mitochondria were attenuated. Baicalin can reduce the production of inflammatory cytokines and ROS in the arteries of AS mice. As a result, a significant reduction in the areas of atherosclerotic plaques was observed in the blood vessels of the mice (Duewell et al., 2010). Junyuan Li et al. established an *in vitro* model of HT-22 cells and found that the expression of NLRP3, thioredoxin interacting protein (TXNIP), and IL-1β was significantly reduced after treatment with 16 μM baicalin (Li et al., 2023). This finding suggested that baicalin can attenuate the damage caused by glutamate-induced oxidation via the NLRP3 inflammasome.

Many findings suggest that baicalin can inhibit pyroptosis (Bai et al., 2023; Liu et al., 2024). In a tuberculosis model, baicalin inhibited the NF-κB pathway and prevented NLRP3 inflammasome activation. Moreover, baicalin downregulated the expression of TXNIP, an activator of the NLRP3 inflammasome, by inhibiting the activation of the PERK/eIF2α pathway. This process resulted in a reduction in pyroptosis in *Mycobacterium* tuberculosis-infected macrophages. Inhibition of the interaction between TXNIP and NLRP3 is one of the pathways by which baicalin reduces focal macrophage pyroptosis (Fu et al., 2021). In addition, recent studies have shown that METTL3 can inhibit ox-LDL-induced macrophage pyroptosis to protect against CVDs (Shu et al., 2021). Conversely, baicalin has a significant regulatory effect on METTL3 (Jiang et al., 2022). Therefore, inhibiting macrophage pyroptosis through METTL3 may be a potential therapeutic strategy for baicalin. The regulation of NLRP3 by baicalin *in vivo* directly reduced inflammatory factor levels and prevented the adverse effects caused by macrophage and EC pyroptosis. The regulation of upstream and downstream effector molecules of NLRP3 is expected to be an important modality for baicalin in the treatment of inflammation-related CVDs.

In summary, the addition of 0–150 μM baicalin to an *in vitro* model effectively inhibited the inflammatory pathway in a dose-dependent manner, reducing the levels of inflammatory factors such as IL-1β, IL-6, and TNF-α. When baicalin was administered at a concentration of 150 μM, the levels of inflammatory factors decreased most significantly, and the inhibition of inflammatory damage was the most effective. In each *in vivo* experimental model, the administered dose was mostly 100 mg/kg/d. However, the relationship between the baicalin concentration and time of administration has not been described in the literature, and further studies are needed to determine the optimal length of drug residence in in vitro models when baicalin is applied *in vivo*.

### 2.2 Antioxidation

**FIGURE 4:**
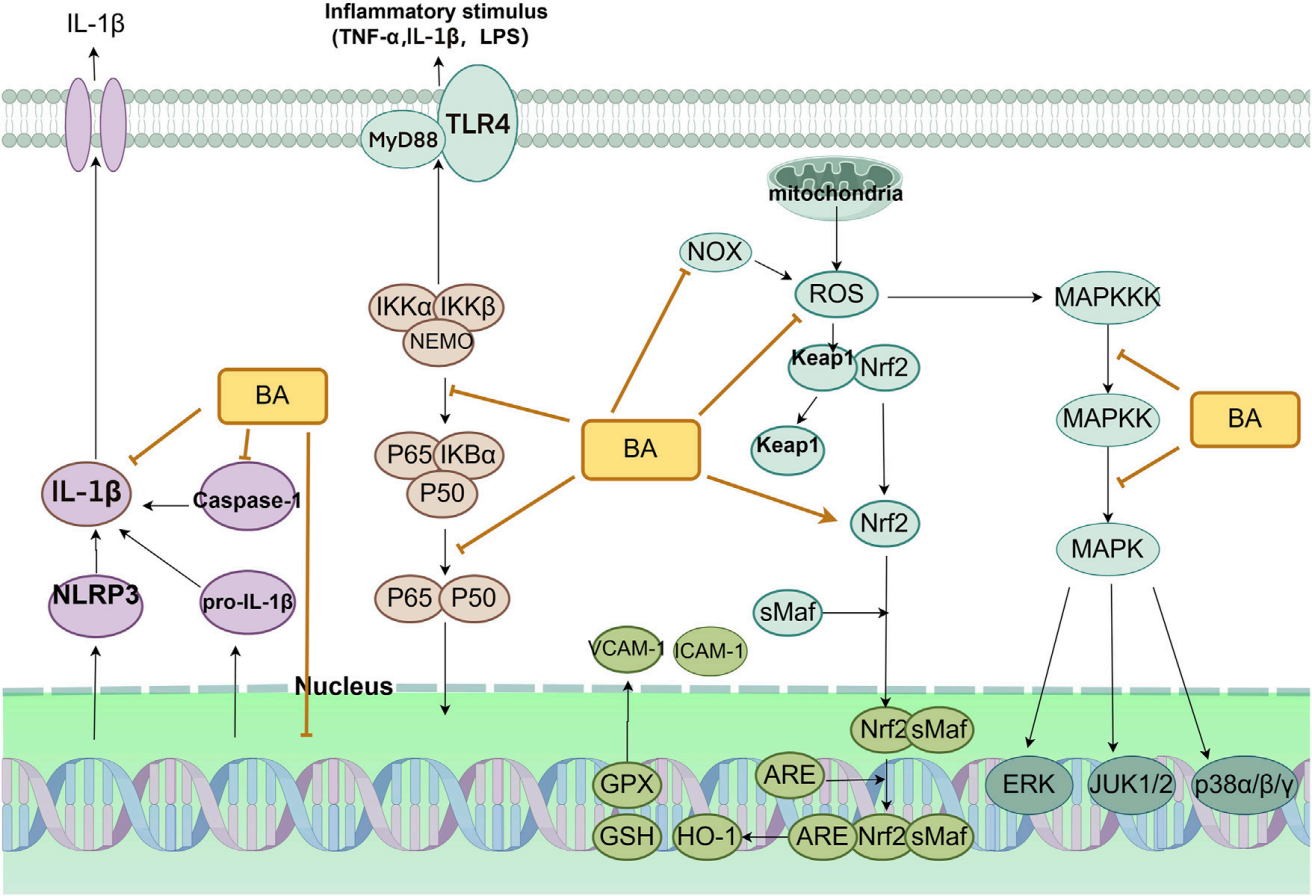
Primary anti-inflammatory and antioxidant mechanism of baicalin in the treatment of CVDs. Abbreviations: baicalin (BA), nicotinamide adenine dinucleotide oxidase (NOX), reactive oxygen species (ROS), heme oxygenase 1 (HO-1), glutathione (GSH), glutathione peroxidase (GPX), vascular cell adhesion molecule-1 (VCAM-1), intercellular adhesion molecule 1 (ICAM-1), toll-like receptor 4 (TLR4), myeloid differentiation primary response protein 88 (MyD88), tumor necrosis factor-α (TNF-α), lipopolysaccharide (LPS), NLR family pyrin domain containing 3 (NLRP3), mitogen-activated protein kinase (MAPK), MAPK kinase (MAPKK), MAPK kinase kinase (MAPKKK), extracellular-regulated kinase (ERK), c-Jun N-terminal kinase (JUK).

Oxidative stress (OS) refers to the disruption of the equilibrium between pro-oxidant and antioxidant defense mechanisms caused by the presence of malondialdehyde (MDA), nitrogen species, and an imbalance in antioxidant defense mechanisms (Sinha and Dabla, 2015). It indirectly destroys cells by causing inflammation and directly affects vascular endothelial function. Hence, a focus on mitigating oxidative stress is crucial when managing CVDs (Steven et al., 2019). Antioxidant enzymes protect against cellular damage caused by ROS. They mainly consist of superoxide dismutase (SOD) and glutathione peroxidase (GSH-px). MDA is a product of unsaturated lipid degradation caused by ROS and is commonly used as a biomarker of oxidative stress. Disruption of the balance between ROS production and removal can result in oxidative stress, which can damage cells and tissues. Since oxidative stress and inflammatory injury are often linked, we summarize the mechanisms involved below (Figure 4).

Recent investigations have shown that baicalin can mitigate oxidative stress in blood vessels by activating the Nrf2 signaling pathway (Fujiwara et al., 2023). The body’s predominant endogenous antioxidant signaling pathway is the Nrf2/Keap1/ARE pathway (Ucar et al., 2021). Nrf2 is an integral part of the CNC family of transcription factors. It is indispensable for regulating the cellular response to oxidative stress by activating corresponding antioxidant elements (Yamamoto et al., 2018). During oxidative stress, the dissociation of Nrf2 from Keap1 is induced. Nrf2 then moves into the nucleus and binds to the sMaf protein, creating a transcriptionally active heterodimer. The Nrf2-sMaf heterodimer recognizes AREs and induces gene transcription, which leads to the activation of an array of antioxidant, protective genes downstream. It also activates corresponding antioxidant enzymes, including heme oxygenase-1 (HO-1) and glutathione S-transferase (GST), among others. This process, in turn, triggers a protective antioxidant response to combat oxidative stress (Bellezza et al., 2018). Insufficient levels of Nrf2 result in heightened levels of oxidative stress, an altered proinflammatory phenotype of vascular endothelial cells (ECs), and increased EC apoptosis (de Oliveira et al., 2019; Hwang et al., 2022). This imbalance impairs vascular endothelial function, impairing cell proliferation and reducing cell migration. Before monitoring the cells, Gen Chen et al. cultivated HUVECs for 72 h in either high-glucose (HG, 33 mM) or normal-glucose (NG, 5.5 mM) medium and added baicalin. Baicalin restored the activity of the Nrf2 pathway in aortic vessels after hyperglycemic injury. At 50 μM, baicalin significantly inhibited oxidative and inflammatory damage in the vasculature (Chen et al., 2018). The increase in the expression of the Nrf2 target genes HO-1, NQO1, NQO2, and SOD and the decrease in the expression of the proinflammatory cytokines IL-6, IL-8, and TNF-a may all be related to the activation of the AKT/GSK3β/Fyn pathway to affect the Nrf2 pathway. In addition, in an animal model of AS, researchers found that baicalin effectively reduced MDA levels by inhibiting the NF-κB and p38 MAPK signaling pathways while increasing the expression of CAT, GSH-Px, and SOD (Wu Y. et al., 2018). However, these studies did not specify the link between the baicalin-regulated NF-κB and p38 MAPK signaling pathways in the pathological process of atherosclerotic plaques, and in-depth studies should be conducted.

Blocking NADPH oxidase (NOX) activity can effectively reduce ROS production and alleviate oxidative stress. NOX is the primary catalytic enzyme for ROS in cardiovascular cells. ROS overproduction leads to hyperactive oxidative stress, which in turn triggers sympathetic nervous system activity and an increase in blood pressure (Petit-Hartlein et al., 2024). Baicalin diminishes NADPH oxidase enzyme activity, decreasing ROS production and preventing oxidative stress from damaging human ECs (Tsai et al., 2021). The results of a network pharmacology study by Mingshuang Li et al. showed that NOX4 is a target of baicalin. Based on this conclusion, they conducted an in-depth study in an endothelial cell model and found that a baicalin intervention resulted in an increase in NOX4 and eNOS levels and a decrease in ROS generation in HAECs. When NOX4 was inhibited, the inhibitory effect of baicalin on ROS generation was eliminated (Li and Ren, 2022). The powerful antioxidant function of baicalin suggests baicalin is a promising anti-atherosclerosis agent.

In summary, in an *in vitro* model, 0–50 μM baicalin was effective at increasing the activity of antioxidant enzymes. In an *in vivo* model, the administration of 50 mg/kg or 100 mg/kg baicalin significantly reduced the damage caused by oxidative stress. However, slight cytotoxicity was observed at concentrations up to 60 μM, and significant cytotoxicity was observed at 200 μM. Therefore, although the therapeutic effect of baicalin is dose-dependent, the dose of baicalin should be limited from a safety point of view.

### 2.3 Inhibition of EC damage and apoptosis

The vascular endothelium is a crucial cell layer involved in metabolic processes, immune activity, and adhesion molecule expression during inflammation. EC apoptosis occurs primarily in the early stages of AS. It leads to plaque regression and destabilization, which can compromise endothelial integrity and cause CVDs (Caland et al., 2019). EC apoptosis is regulated mainly by cysteine asparaginase. Caspase-3 cleavage activates the cysteine asparaginase cascade, which is a significant hallmark of apoptosis.

The PKCδ/p53 signaling pathway efficiently initiates the cysteine asparaginase cascade, which induces apoptosis. PKCs belong to the AGC family of serine/threonine protein kinases (Black and Black, 2012). Ten different isoforms of PKCs have been identified: classic, nonclassical, and novel. PKCδ is a novel isoform that has various biological functions and can regulate proliferation, differentiation, and apoptosis. Moreover, p53 is an essential substrate of PKCδ. Upon stimulation, PKCδ phosphorylates and activates p53, causing apoptosis through the cleavage of caspase-3 and stimulation of the cysteine asparaginase cascade (Dashzeveg and Yoshida, 2016). Human arterial endothelial cells (HAECs) were exposed to a hypoxia/reoxygenation (H/R) environment, as ECs are susceptible to damage caused by H/R, to examine the defensive effect of baicalin on ECs. The results revealed a significant reduction in PKCδ and p53 phosphorylation following baicalin administration. This effect was dose-dependent, with 10, 20, and 40 μmol/L baicalin progressively inhibiting PKCδ and p53 phosphorylation. When the PKCδ/p53 apoptotic signaling pathway was inhibited in HAECs subjected to H/R, the activation of the cysteoaspartase cascade reaction was also blocked, resulting in an obvious antiapoptotic effect (Shou et al., 2017). This finding suggested that baicalin may modulate the PKCδ/p53 signaling pathway to prevent the activation of the cysteine asparaginase cascade and reduce apoptosis.

The receptor-interacting protein (RIP) family is essential for the regulation of apoptosis and necrosis. RIP1 can activate signaling pathways related to programmed cell death. Jiannan Bai et al. reported that baicalin protected cardiac microvascular endothelial cells (CMECs) from I/R injury in a rat model. After treating CMECs with 6.3, 25, or 100 μg/mL baicalin *in vitro*, RIP1, RIP3, and p-MLKL protein expression were inhibited in the 25 μg/mL baicalin group, and CMEC apoptosis was prevented. Moreover, researchers found that the area of myocardial infarction was significantly reduced, and cardiac function was significantly improved in the groups treated with 30 or 100 mg/kg/day baicalin after the rats were administered 10, 30 or 100 mg/kg/day baicalin (Bai et al., 2019). The researchers found that 25 μg/mL baicalin reduced I/R damage in CMECs by altering the expression of the RIP1, RIP3, and p-MLKL proteins. The highest suppression of apoptosis occurred after treatment with a concentration of 100 μg/mL.

Activation of the NF-κB pathway is another significant mechanism for generating damage and death in ECs. Cytokines, including TNF-α, IL-1β, NF-κB, and IκB, as well as ROS, translocate into the nucleus to promote the transcription of target genes when cells are stimulated by hypoxia (Wu and Yan, 2024). Additionally, a surge in the phosphorylation of p65 and activation of NF-κB was observed as the occurrence of thrombin-induced cell death increased in HUVECs. These findings support those of prior research and indicate that NF-κB is crucial for programmed cell death in ECs. The expression of p-P65 in HUVECs was attenuated in a dose-dependent manner after treatment with 50, 100, or 150 μM baicalin. Additionally, baicalin can reduce EC apoptosis by inhibiting PAR-1 expression and blocking the activation of the ERK signaling pathway. This pathway also has pivotal functions in inflammation and apoptosis. A notable increase in EC apoptosis was observed following the activation of ERK1/2, and the expression of PAR-1 facilitated cell apoptosis through a mechanism relying on caspase-3. Baicalin directly inhibits the overexpression of PAR-1 and limits the extent of caspase-3 protein cleavage. Furthermore, the activation of ERK1/2 facilitates thrombin-induced PAR-1 production. Therefore, inhibition of the ERK signaling pathway impedes the activation of the cysteine cascade (Zhang et al., 2019). Therefore, the regulation of the ERK signaling pathway by baicalin not only directly reduces apoptosis but also indirectly reduces apoptosis by inhibiting PAR-1 production and blocking the activation of the cysteine cascade through the inhibition of ERK1/2.

By controlling programmed cell death and mitigating cellular damage induced by oxidation and inflammation, baicalin successfully reduced endothelial cell apoptosis. In the HUVEC model, baicalin modulates both the ERK1/2 pathway and the NF-κB pathway to reduce endothelial cell apoptosis through both anti-inflammatory and antioxidant mechanisms. The minimum active concentration required to exert this effect was 50 μM, and the best effect was achieved with a concentration of 150 μM. This dose-dependent effect was also observed in experiments in which baicalin modulated the RIP family to reduce endothelial cell apoptosis.

### 2.4 Impact on innate immunity

During the activation of innate immunity in the initial stages of AS, damage to the endothelium induces the release of cytokines that attract monocytes and leukocytes to the site of inflammation (Gisterå and Hansson, 2017). Subsequently, monocytes and leukocytes that transform into macrophages mediate the oxidative modification of subendothelial LDL to form ox-LDL. Additionally, the type A scavenger receptor (SR-A) promotes the engulfment of ox-LDL by macrophages, which results in the accumulation of lipids inside cells and the eventual production of foam cells (Yuan et al., 2012; Chistiakov et al., 2017). During this process, macrophage particles contribute to ROS generation, cause macrophage apoptosis, and generate foam cells. Thus, the specific timing of macrophage apoptosis exerts distinct impacts on the development of AS. Early-stage apoptosis is conducive to inhibiting foam cell formation. In advanced atherosclerotic plaques, macrophage apoptosis contributes to the formation of necrotic cores in a timely manner and triggers NLRP3 activation to accelerate AS development.

Macrophage polarization is a process by which macrophages differentiate into two distinct types, proinflammatory (M1) and anti-inflammatory (M2) macrophages, in response to various stimuli. M1 macrophages release proinflammatory cytokines, including IL-6, IL-1β, and TNF-α, while M2 macrophages release anti-inflammatory cytokines, such as IL-10 and Arg-1. A study revealed that M2 macrophages can induce the death of M1 macrophages (Wan et al., 2014). Hence, varying polarization orientations have distinct impacts on the progression of CVDs. Specifically, an increase in the proportion of the M1 subtype can facilitate the development of AS. Through ROS and RASS mechanisms, M1 macrophages generate inflammatory cytokines that induce detrimental effects.

#### 2.4.1 Cholesterol efflux and apoptosis

The membrane transporter proteins ATP-binding cassette subfamily A member 1ABCA1 and ABCG1 are regulated by PPARs and LXR-α/β. As an important receptor for regulating plasma HDL and intracellular cholesterol levels, it is involved in macrophage cholesterol efflux and mediates intracellular cholesterol efflux to apolipoprotein A1 (ApoA1). An enhancement of arterial macrophage ABCA1 activity in a mouse model of AS promoted cholesterol efflux onto ApoA1 to inhibit foam cell formation (Tsou et al., 2014). ABCA1 also stimulates macrophage phagocytosis and particle production, thereby contributing to the formation of atherosclerotic plaques (Fond et al., 2015). LXRα is a target gene of PPAR-γ and is a key regulator of reverse cholesterol transport (RCT). The metabolic cascade involving PPAR-γ and LXRα efficiently regulates the function of downstream genes to increase the efflux of cholesterol from macrophages (Marasinghe et al., 2024). Baicalin treatment resulted in a notable decrease in total cholesterol (TC) and LDL levels and stimulated the expression of PPARγ and LXRα in THP-1 macrophages located in the carotid arteries of rabbits with AS. Furthermore, it facilitated an increase in the mRNA and protein expression levels of the cholesterol efflux transporters ABCA1 and ABCG1 in macrophages (He et al., 2016). Baicalin may increase the expression of cholesterol efflux transporter proteins by stimulating the expression of PPARγ and LXRα to reduce foam cell formation.

The scavenger receptor (SR) pathway is the main pathway mediating the cellular uptake of ox-LDL. Its expression is a key factor influencing foam cell formation. Scavenger receptor class B type I (SR-BI) is an HDL receptor belonging to the CD36 protein superfamily that mediates cholesterol interflow between cells and HDL. PPAR-γ induces SR-BI expression and facilitates the excretion of cholesterol (Yu et al., 2013). Cyanide stimulates PPAR-γ, which leads to increases in the expression of PPARγ and LXRα, hence promoting cholesterol efflux. Further evidence suggested that SR-BI plays a key role in the elimination of cholesterol induced by baicalin. Renchao Yu et al. chose different concentrations of baicalin to treat cells, and the results showed that 50 μM baicalin had the greatest effect on promoting SR-BI expression after 48 h. Further experiments showed that 50 μM baicalin was effective at promoting SR-BI expression at 0, 6, 12, 24, and 48 h, and SR-BI expression increased at 12 h and peaked at 24–48 h. The results showed that 50 μM baicalin was the most effective dose at promoting SR-BI expression after 48 h. Similarly, the expression of LXRα and PPAR-γ peaked at 12 h after treatment with 50 μM baicalin, with a significant increase in cholesterol efflux. This effect was suppressed when BLT-1, a specific inhibitor of SR-BI, and SR-BI siRNA were used (Yu et al., 2016). Therefore, researchers have concluded that baicalin promotes cholesterol efflux from THP-1 macrophages by activating the PPAR-γ/LXRα/SR-BI pathway.

#### 2.4.2 Macrophage polarization

Two distinct pathways for macrophage polarization have been identified. Macrophages stimulated by type 1 T helper cytokines such as IFN-α and LPS are polarized toward the M1 phenotype. Macrophages of the M1 phenotype can upregulate iNOS, produce IL-1, IL-6. Type 2 T helper cytokines, such as IL-4 and IL-23, induce macrophages to transform into the M2 phenotype. M2 macrophages upregulate Arg-1, Fizz1, and Ym-1, producing IL-10 and TGF-β. Macrophages polarized in different directions also have different effects on CVDs (Ma et al., 2024). Previous studies of colitis have shown the effect of baicalin on macrophage polarization. Baicalin potently influences the direction of macrophage polarization, encouraging M2 polarization. This process is achieved by suppressing the expression of the IRF5 protein and increasing the expression of the IRF4 protein in lamina propria monocytes. Additionally, M1 macrophages repolarize to the M2 phenotype in response to LPS (Zhu et al., 2016). Studies have indicated that MERTK is essential for macrophages to trigger apoptosis for phagocytosis (Akalu et al., 2017; Du et al., 2017). In an *in vitro* experiment conducted by Yin-Siew Lai et al., macrophages treated with 50 μM baicalin and 10 ng/mL IL-4 were divided into two groups to observe their polarization. The results of the experiments showed that baicalin not only upregulated the expression levels of IL-10 and its receptor compared to those of IL-4 but also contributed to the polarization of macrophages toward the M2C phenotype by upregulating MERTK and PTX3. Researchers have concluded that this finding reveals the potential feasibility of baicalin for the treatment of AS (Lai et al., 2018). Another study analyzing the cardioprotective effects of baicalin on an I/R model suggested that the increase in the number of M2-type macrophages in damaged myocardial tissue showed a dose-dependent relationship with baicalin. The greatest increase in the number of M2-type macrophages was observed when 120 mg/kg baicalin was administered. In this process, the levels of cleaved capase3, iNOS, and IL-1β were decreased, and the levels of Arg-1 and IL-10 were increased by baicalin treatment (Xu et al., 2020). The specific mechanism may be related to the reduction in the phosphorylation of the JAK2 and STAT3 proteins and the inhibition of the JAK/STAT pathway.

### 2.5 Effects on vascular smooth muscle cells

Vascular smooth muscle cells (VSMCs) are located in the cell walls of blood vessels. Typically, VSMCs produce contractile proteins such as smooth muscle α-actinin (α-SMA), smooth muscle 22α protein (SM22α), and osteopontin (OPN). This process results in a robust ability to contract by encouraging VSMC synthesis. However, when VSMCs are exposed to LPS, they transition from a highly differentiated state to a synthetic state with a decreased degree of differentiation, which has the capacity for reproduction. At that time, the migration and proliferation of cells are dramatically improved (Lacolley et al., 2017; Liu et al., 2017). Overproliferation of VSMCs is one of the leading causes of neointimal thickening and vascular luminal narrowing. The pathological basis of a significant proportion of CVD cases is decreased vascular compliance and increased blood pressure. Therefore, investigating the regulation of excessive VSMC growth and movement is crucial.

#### 2.5.1 Inhibition of VSMC proliferation and migration

Platelet-derived growth factor (PDGF) belongs to the VEGF family. PDGF signaling requires the PDGF receptor (PDGFR), which has two isoforms, PDGFRα and PDGFRβ. Among these proteins, PDGFR-β is centrally expressed in vascular ECs. When injury occurs, ECs secrete abundant PDGF to promote proliferation and migration to repair injury (Bernard et al., 2024). PDGF specifically binds to and activates PDGFR to undergo autophosphorylation, triggering signaling cascades, one of which is the MEK/ERK signaling pathway. ERK1/2 is critical for intracellular signaling in response to an extracellular stimulus. Upstream signals are delivered via the RAS/RAF/MEK/ERK signaling cascade (Wang et al., 2019). Activated ERK is translocated to the nucleus to activate proliferation-related substrates.

Many studies have shown that baicalin inhibits VSMC proliferation by blocking PDGF upregulation (Dong et al., 2010; Li J. et al., 2019). A study using an animal model of carotid balloon injury showed that baicalin effectively inhibited intimal proliferation by affecting the aforementioned process. Cell counts indicated that the proliferative activity of PDGF-stimulated VSMCs tended to decrease with increasing baicalin concentration. Baicalin upregulated the expression of the cell cycle kinase inhibitor p27, preventing its phosphorylation by inhibiting the activation of ERK1/2 and the cell cycle protein E-CDK2 and fully inhibiting the proliferation of VSMCs. This finding points to the possibility that baicalin inhibits PDGF-BB and MEK/ERK to reduce VSMC proliferation (Dong et al., 2010).

The migration of many proliferating VSMCs from the mesentery to the intima is critical for intimal thickening. The migration of VSMCs results in the substantial accumulation of VSMCs in one location, which directly contributes to AS development. The formation of pseudopodia on the front of cells is a characteristic feature of VSMC migration. The formation of anterior pseudopodia is a morphological feature of migrating cells. Cytoskeletal remodeling and related proteins provide the material basis for pseudopodia. SM22, a cytoskeletal protein, can limit plaque growth by inhibiting the phenotypic transformation of VSMCs. The enhancement of VSMC migratory activity is one of the important features of phenotypic transformation. Jiankun Li et al. reported that baicalin plays an important role in the inhibition of PDGF-BB-induced VSMC migration through SM22α. When VSMCs were pretreated with baicalin at a concentration of 70 mg/kg/day for 24 h, SM22α was overexpressed, and PDGF-BB-induced VSMC migration was blocked (Lv et al., 2016). Researchers have concluded that the ability of baicalin to inhibit VSMC proliferation and migration is related to the upregulation of SM22α expression and the blockade of the Ras-Arp2/3 signaling pathway.

#### 2.5.2 Inhibition of VSMC hypercontraction

The renin–angiotensin system (RAS) is an important component of the pathogenesis of hypertension. Renin converts angiotensinogen to angiotensin I (Ang I), which is released from Ang I by angiotensin I-converting enzyme (ACE). Modulation of Ang II is the focus of most current studies on the vasodilatory mechanism of baicalin. Research has shown that baicalin expands blood vessels by hindering the activity of renin and ACE and has the capacity to decrease Ang II levels by converting Ang II into Ang-(1–7) (Deng et al., 2012). This process leads to increased expression of ACE2 and Mas, the receptor for Ang-(1–7). The upregulation of Mas expression facilitates vasodilation and mitigates oxidative stress. By mediating the ACE2/Ang-(1–7)/Mas axis, baicalin positively affects cardiovascular activity (Wei et al., 2015). Ca2+ regulation is another critical factor in the regulatory mechanisms of vascular smooth muscle. Ang II binds to the angiotensin II type 1 receptor (AT1R), which increases the concentration of free calcium ions in the cell. Calcium ions that are not bound attach to calmodulin and stimulate myosin light chain kinase (MLCK), an enzyme that adds a phosphate group to the myosin light chain (MLC). This phosphorylated MLC then attaches to actin, forming a crossbridge. As a result, the myofilaments slide past each other, leading to vasoconstriction. By investigating the mechanism of intracellular Ca2+ modulation in vascular smooth muscle and its relationship with baicalin, researchers have discovered that baicalin reduces aortic constriction by decreasing Ca2+ release through the control of PE and Ang II (Ding et al., 2019). Baicalin inhibits the binding of Ang II to AT1R, hence reducing the release of intracellular calcium in VSMCs. Activation of the MLCK/MLC pathway is suppressed, which leads to the regulation of smooth muscle contraction (Liu et al., 2021). The researchers concluded that the relaxing effect of baicalin on vascular smooth muscle in hypertensive rats provides valid evidence for further clinical studies of baicalin in the treatment of hypertension.

### 2.6 Regulation of coagulation and the fibrinolytic system

Following damage to ECs, a cascade of events occurs involving platelet activation, adhesion, aggregation, and thrombosis. Activated platelets produce proinflammatory factors such as IL-1β and IL-8, and these proinflammatory factors contribute to thrombus formation (Mack et al., 2024). Suppressing platelet activity and implementing anticoagulation measures play crucial roles in halting the progression of AS.

Recent research has indicated that baicalin successfully reduces the TNF-α-induced adhesion and aggregation of platelets on HUVECs. Additionally, it restricts the expression of ICAM-1, TSP-1, and collagen and prevents collagen-induced AKT1 phosphorylation in ECs. Researchers have hypothesized that baicalin may hinder platelet activation by inhibiting AKT-related pathways (Lee et al., 2015b). By assessing alterations in the clotting time, FXa generation, and the PAI-1/t-PA ratio, researchers found that 50 μM baicalin significantly increased the PT and aPTT and hindered fibrin polymerization and platelet aggregation. Furthermore, baicalin decreased the synthesis of PAI-1, leading to a reduction in the PAI-1/t-PA ratio, which indicates thrombolytic action. A series of data suggests that baicalin impedes thrombus formation through a mechanism that inhibits FXa and thrombin production (Lee et al., 2015b). Unsurprisingly, baicalin can slow the progression of CVDs by preventing AS through decreases in prothrombin, proinflammatory factor, and anticoagulant pathway activity. However, whether the anticoagulant properties of baicalin may exacerbate the progression of coagulation disorders or potentially exhibit bidirectional regulation remains unconfirmed. Therefore, adequate weight and consideration should be given when administering baicalin to patients with coagulation abnormalities.

## 3 Mechanisms of action of baicalin in the heart

**FIGURE 5:**
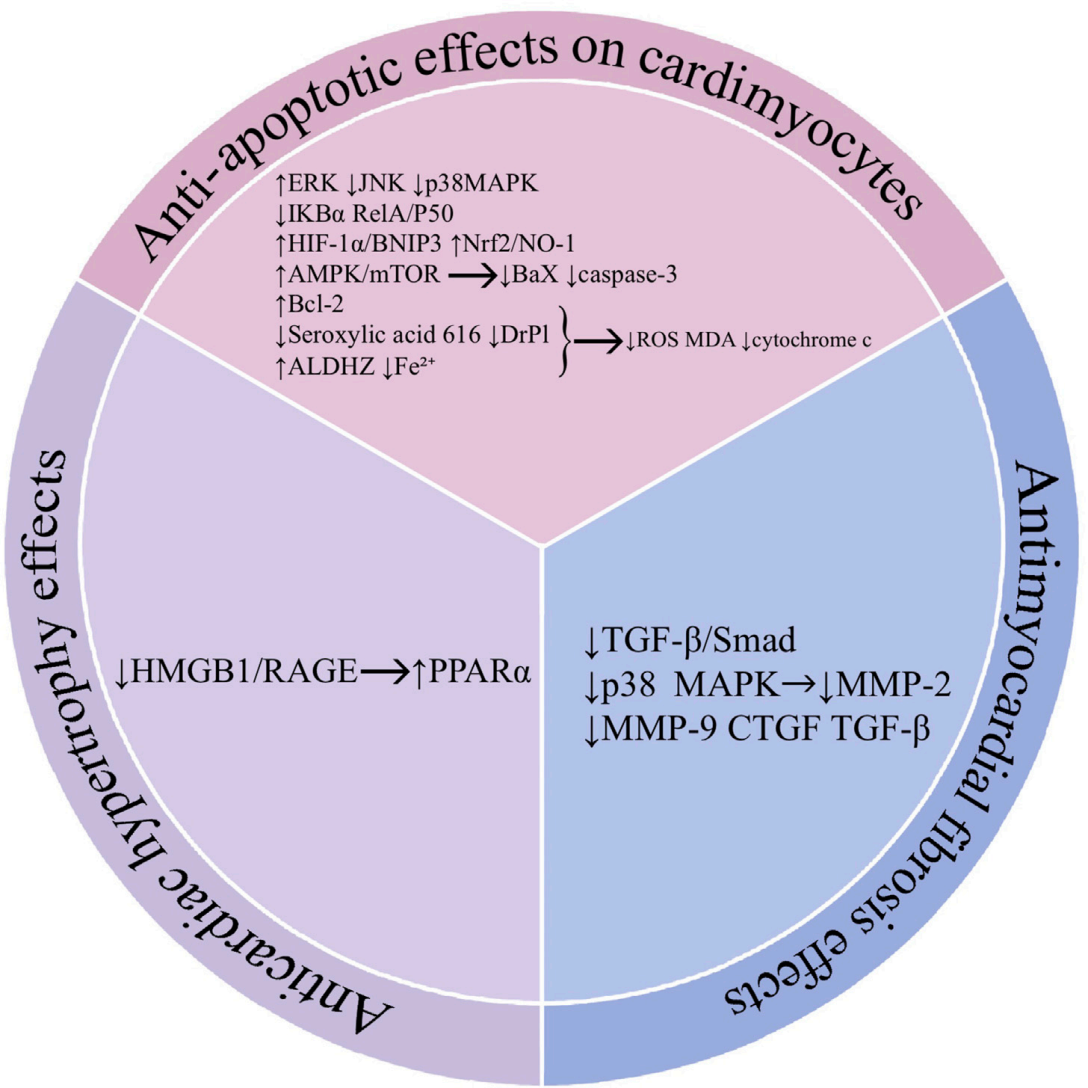
Mechanisms of action of baicalin in the heart. Abbreviations: Hypoxia-inducible factor 1 (HIF-1α), c-Jun N-terminal kinase (JNK), BCL2 interacting protein 3 (BNIP3), dynamin-related protein 1 (Drp1), aldehyde dehydrogenase 2 (ALDH2), adenosine 5-monophosphate (AMP)-activated protein kinase (AMPK), mammalian target of rapamycin (mTOR), B-cell lymphoma-2 (Bcl-2), BCL2 associated X (Bax), high mobility group protein (HMGB1), receptor for advanced glycation end products (RAGE), proliferator-activated receptor α (PPARα), transforming growth factor-β (TGF-β), *drosophila* mothers against decapentaplegic protein (Smad), metalloproteinases-2 (MMP-2), metalloproteinases-9 (MMP-9), connective tissue growth factor (CTGF).

In the heart, cardiomyocyte apoptosis leads to a compensatory enhancement of the function of surviving cardiomyocytes and adaptive hypertrophy of cardiomyocytes. As the degree of hypertrophy increases and cardiomyocytes undergo further apoptosis, the overall contractility of the myocardium decreases, resulting in the development of myocardial fibrosis. This change can lead to a continuous decline in cardiac function and serious adverse effects on the heart. Therefore, we summarize the mechanism of action of baicalin from the three aspects of cardiomyocyte apoptosis, cardiomyocyte hypertrophy, and cardiomyocyte fibrosis (Figure 5).

### 3.1 Anti-apoptotic effects on cardiomyocytes

Cardiomyocyte apoptosis is the programmed death of cardiomyocytes. Myocardial ischemia/reperfusion (I/R) injury is closely related to the occurrence of cardiomyocyte apoptosis. This model has been widely used in the majority of investigations into the mechanism by which baicalin affects the heart. I/R injury results in decreased mitochondrial activity, excess ROS generation, and a rapid increase in oxidative stress. This process ultimately causes the death of cardiomyocytes (Xiang et al., 2024). Simultaneous activation of the endoplasmic reticulum during this period also triggers apoptosis in cardiomyocytes, which rely on the endoplasmic reticulum for their function. Apoptotic cardiomyocytes initiate an inflammatory reaction that exacerbates the progression of apoptosis (Del Re et al., 2019). Proapoptotic proteins play vital roles in the process of apoptosis. Among these proteins, the caspase family and the Bcl-2 family are the most frequently investigated. Caspase-3 is a key protease in the apoptotic cascade, and Bcl-2 and Bax belong to the Bcl-2 family. Bcl-2 is an inhibitor of apoptosis, whereas Bax activates caspase-3 by allowing cytochrome c to cross the mitochondrial membrane to promote apoptosis (Julien and Wells, 2017). Based on the major processes involved in I/R injury, antioxidant and anti-inflammatory agents might be effective strategies to fight apoptosis in cardiomyocytes.

The MAPK pathway and NF-κB pathway are classic pathways involved in the induction of inflammation. Research has shown that blocking the ERK pathway intensifies the process of apoptosis in cardiomyocytes, which is in direct contrast to the effects of blocking the JNK and p38 pathways. During the antiapoptotic process in cardiomyocytes, rat cardiomyocytes treated with baicalin exhibited increased p-ERK expression and decreased p-JNK and p-p38 expression. This approach significantly improved the hemodynamic parameters of the left ventricle and myocardial I/R injury in the rat myocardium (Liu et al., 2013). Another study suggested that baicalin-mediated activation of the ERK pathway may be related to CaSR (Liu et al., 2020). The modulation of calcium channels by baicalin activates the ERK pathway to safeguard against cardiomyocyte apoptosis. Furthermore, it directly inhibits LPS-induced apoptosis by lowering the calcium overload mediated by STIM1 (Li M. F. et al., 2019). In an *in vitro* I/R model, pretreatment of cardiomyocytes with 10 μM baicalin significantly inhibited the nuclear translocation of NF-κB, decreased IL-6 levels, and increased IL-10 levels. These changes protected cardiomyocytes from damage caused by inflammation and oxidative stress (Lin et al., 2010). Additional experimental findings demonstrated that by modifying the TLR4/IκBα/NFκB pathway, 100 mg/kg baicalin administered to rats dramatically prevented IκBα phosphorylation and decreased cardiomyocyte apoptosis caused by inflammation and oxidative stress (Feng et al., 2022).

Baicalin protects cardiomyocytes from apoptosis through its antioxidant effects. HIF-1α is often expressed under hypoxic conditions and affects the expression of the downstream gene BNIP3. Increasing the activity of the HIF-1α/BNIP3 pathway has been reported to safeguard against MIRI. Baicalin promotes the expression of HIF-1α and BNIP3 in cardiomyocytes through the activation of the Nrf2/HO-1 pathway (Yu et al., 2019). The attenuation of cardiomyocyte apoptosis by the HIF-1α/BNIP3 pathway is related to its ability to mediate mitochondrial autophagy (Zhang Y. N. et al., 2022). Mitochondrial dysfunction is caused by oxidation, which in turn leads to cardiomyocyte damage via oxidation. Mitochondrial fission, which is mainly controlled by Drp1 activity, often occurs in the heart after I/R injury. Excessive mitochondrial fission results in impaired energy metabolism, elevated ROS levels, and the release of proapoptotic factors from cardiomyocytes. Baicalin effectively reduced cardiomyocyte apoptosis by inhibiting serine 616 phosphorylation and Drp1 translocation-mediated mitochondrial fission, hindering ROS production and cytochrome c release in cardiomyocytes (Wu et al., 2021). Mitochondrial aldehyde dehydrogenase 2 (ALDH2) inhibits oxidative stress, and its absence results in mitochondrial dysfunction. Research has shown that baicalin effectively suppresses the generation of MDA and ROS by enhancing ALDH2 activity in H9c2 cardiomyocytes. In addition, it promotes the expression of the Bcl-2 protein while reducing the activity of the caspase-3, cytochrome c, and Bax proteins. This activation successfully suppresses H/R-induced apoptosis and oxidative stress in cardiomyocytes (Jiang et al., 2018). Ferroptosis is programmed cell death caused by iron-dependent oxidative stress. Lipid oxidation is closely related to ferroptosis. In recent years, several scholars have focused on the link between ferroptosis and baicalin-mediated protection of cardiomyocytes by performing in-depth studies. Baicalin decreased the accumulation of Fe^2+^ and decreased the formation of ROS and lipid peroxidation in cardiomyocytes. NCOA4 treatment decreased iron accumulation in cells by minimizing the activation of TfR1 signaling and ferritin-induced autophagy (Fan et al., 2021). This difference may be related to the reversal of ACSL4 expression by baicalin.

In addition, baicalin affected the apoptosis of vascular endothelial cells by regulating AMPK (Zhang L. et al., 2023). Recent studies have also confirmed that baicalin can attenuate angiotensin II-induced apoptosis and autophagy in cardiomyocytes by modulating the AMPK/mTOR pathway, reversing the upregulation of Bax, caspase 3, and cleaved cysteine asparaginase nine levels and the downregulation of Bcl-2 (Cheng et al., 2024). This finding suggested that the role of baicalin in attenuating apoptosis may be related to its effect on the mechanism regulating cellular energy homeostasis.

In summary, most of the *in vitro* studies on the antiapoptotic mechanism of baicalin in cardiomyocytes used H9C2 cells, the drug dose used ranged from 10 to 100 μM, and the treatment time ranged from 12 h to 24 h. A correlation was observed between the selected dose and the time; for example, the duration of treatment with 10 μM baicalin was mostly 12 h. During the process of baicalin treatment, caspase-3 and Bax protein expression were downregulated, and Bcl-2 expression increased in H9C2 cells, indicating the good antiapoptotic properties of baicalin. However, the experiment did not mention the relationship between time and the efficacy of baicalin, and the data should be improved by performing in-depth experiments.

### 3.2 Anticardiac hypertrophy effects

Pathologic cardiac hypertrophy is a compensatory enlargement of cardiomyocytes caused by genetics or a long-term increase in cardiac afterload. During the compensatory process, hypertrophied cardiomyocytes have an increased oxygen demand and require additional energy. Cardiomyocytes obtain energy from two processes, lipid and glucose metabolism, with fatty acid oxidation (FAO) contributing to approximately 70% of ATP production. During pathological conditions, the energy metabolism of cardiac mitochondria is disrupted, resulting in a change from relying on lipid metabolism to primarily depending on glucose metabolism. Dysregulation of energy metabolism inside mitochondria contributes to CVDs (Cai et al., 2019).

Peroxisome proliferator-activated receptor α (PPARα) is a central transcriptional regulator of fatty acid metabolism-related enzymes in the heart. It can promote the homeostasis of myocardial energy metabolism by regulating downstream genes to positively regulate FA oxidation and negatively regulate glucose utilization in cardiomyocytes (Cai et al., 2019). Previous studies have shown that PPARα has a negative regulatory role in the development of pathological cardiac hypertrophy (Kar and Bandyopadhyay, 2018). Downregulation of PPARα activity and expression during cardiac hypertrophy inhibits CPT-1 expression, which reduces the ability of the myocardium to maintain energy metabolic homeostasis. In contrast, PPARα agonists prevent myocardial mitochondrial damage and ameliorate cardiac hypertrophy (Jen et al., 2017). Previous research on lipid metabolism has shown that baicalin successfully stimulates PPARα, which may be the primary mechanism that counteracts cardiomyocyte hypertrophy (Yu et al., 2016). Studies have shown that baicalin induces PPARα and PPARβ/δ expression, attenuates pressure overload-induced cardiac hypertrophy, and inhibits ventricular remodeling in both mouse models and H9C2 cells (Zhang et al., 2017). According to Zhenjie Chen and colleagues, the highest level of PPARα activation and HO1 expression was achieved with 30 mg/kg/d of baicalin (Chen and Wang, 2017). The highest possible safe dose of baicalin inhibits the HMGB1/RAGE pathway to promote PPARγ activation and significantly inhibits cardiac hypertrophy.

### 3.3 Antimyocardial fibrosis effects

Myocardial fibrosis (MF) is a pathological phenomenon characterized by the excessive proliferation of interstitial fibroblasts and the significant accumulation of extracardiac matrix (ECM) (Umbarkar et al., 2021). This process leads to structural alterations in cardiac tissue. When cardiomyocytes are damaged, they undergo degeneration, necrosis, apoptosis, structural changes, and metabolic disorders. Immune cells, especially macrophages, release active growth factors that stimulate the activation and multiplication of cardiac fibroblasts. After cardiomyocyte damage, fibrogenic mediators such as transforming growth factor-β (TGF-β) and PDGF can induce fibroblast differentiation and promote the progression of myocardial fibrosis (Maruyama and Imanaka-Yoshida, 2022). The main feature of these cells is a disproportionate and disordered arrangement of collagen components in cardiomyocytes. This change causes myocardial contractile dysfunction and disrupts the coordination of myocardial excitation–contraction coupling. Cellular matrix metalloproteinases (MMPs) affect the process of myocardial fibrosis by degrading extracellular matrix proteins (Chiao et al., 2012). The continued progression of myocardial fibrosis can lead to arrhythmias, sudden death, and CVDs.

Inhibition of AMPK to suppress the Ang II-stimulated TGF-β/Smad signaling pathway may be the mechanism by which baicalin hinders the onset of myocardial fibrosis. This process hinders the cell development stimulated by Ang II, as well as the production of collagen, fibronectin (FN), and connective tissue growth factor (CTGF) proteins in cardiac fibroblasts (Xiao et al., 2018). Baicalin also inhibits myocardial fibrosis by directly reducing the expression of the fibrosis-related factors MMP-2, MMP-9, CTGF, and TGF-β in cardiomyocytes (Dai et al., 2017). Compared with chronic hypoxic rats, hypoxic rats injected with 30 mg/kg baicalin exhibited favorable antimyocardial fibrosis properties. MMP-9 protein expression, which was elevated by hypoxia in the walls of small pulmonary arteries and in tissue homogenates, was reduced by baicalin treatment, and HE staining showed that baicalin attenuated right ventricular hypertrophy and myocardial disorganization (Yan et al., 2016). In a pressure-overload mouse model, baicalin decreased the elevations in collagen type I, collagen type III, and CTGF mRNA expression levels and attenuated the development of cardiac fibrosis (Zhang et al., 2017). The experimental results suggest that the mechanism by which baicalin inhibits cardiomyocyte fibrosis is related to a reduction in MMP-9 production through the regulation of the p38 MAPK pathway.

The above *in vivo* and *in vitro* experiments in rats and H9C2 cells suggest that baicalin affects the process of myocardial fibrosis by regulating fibrosis-related pathways such as the TGF-β and p38 MAPK pathways and stromal cell proteins. The inhibition of Ang II by baicalin at concentrations up to 100 μM was both effective and safe, but at concentrations up to 200 μM, a decrease in cellular activity was observed. In these *in vivo* experiments, the researchers administered baicalin to rats at 30 mg/kg and to mice at 100 mg/kg; however, they did not discuss the effect of time on the effects of the drug, nor did they examine the effects of other doses of baicalin on the results of the experiments, which should be supplemented to enhance the rigor of the study of the mechanism of baicalin.

## 4 Safety and toxicological studies of baicalin

As the pharmacological effects of baicalin and baicalin glycoside have been explored in depth, their clinical safety and adverse effects have also received attention. According to the National Institutes of Health official guidance, the use of *Scutellaria* “has been implicated in rare instances of clinically apparent liver injury” and “the onset of symptoms and jaundice occurred within 6–24 weeks of starting skullcap, and the serum enzyme pattern was typically hepatocellular”, with “marked increases in serum alanine transaminase, aspartate transaminase, alkaline phosphatase and bilirubin levels.” (https://livertox.nih.gov/Skullcap.htm) Recently, Naohiro Oshima et al. addressed the issue of the relationship between an *S. baicalensis* extract and liver dysfunction. The results of *in vitro* experiments suggested that after baicalin removal, the *S. baicalensis* extract was not cytotoxic, and the hepatocytotoxicity of the *S. baicalensis* extract varied with the concentration of baicalin. The number of cells decreased significantly when the concentration of baicalin reached 250 μg/mL, and decreasing the baicalin concentration could effectively control the aggravation of toxicity (Oshima et al., 2024). Other studies have indicated that gut microbes can attenuate the toxicity of baicalin to hepatocytes (Zhang S. et al., 2022). However, the results of previous trials concluded that high-dose (800–1,600 mg/kg) baicalin may pose a risk of nephrotoxicity in rats and that it does not affect liver function (Cai et al., 2017). Clinical data also show that in randomized, double-blind trials of single oral doses of 100–2,800 mg of baicalin, none of the 72 healthy adults experienced any adverse effects, and no signs of liver or kidney toxicity were detected (Li et al., 2014). Numerous findings have confirmed the hepatoprotective effects of baicalin (Yang et al., 2021; Wang R. et al., 2023).

Therefore, based on the results of the current study, whether baicalin is hepatotoxic in humans remains uncertain. The mechanisms involved in causing hepatotoxicity have also not been fully clarified. Thus, more research is needed to elucidate these issues.

## 5 Conclusion and future perspectives

Cardiovascular disease is currently the leading cause of death. Its onset is related to a variety of factors, such as smoking and obesity. Based on the complexity of the condition of CVDs, a variety of therapeutic strategies for CVDs have been proposed. One of the most often utilized regimens for CVDs treatment is medication combination, which reduces the single drug dose while improving patient tolerance and compliance. Clinical research has demonstrated the efficacy of Chinese medicine as an alternative therapy for CVDs when combined with western medications. Tanshinone IIA combined with atorvastatin effectively attenuated atherosclerosis in rats. When nimodipine and total saponins from Panax ginseng were taken together, the damage from hypertensive cerebral hemorrhage was lessened. In previous studies, Baicalin, an extract from *S. baicalensis*, has demonstrated anti-inflammatory, antioxidant, and other cardiovascular system benefits. This suggests that baicalin has strong potential to be one of the options for combination medications. Studies on baicalin in combination for the treatment of CVDs have not, however, been conducted. Therefore, the strategy of combining baicalin needs to be further studied.

We summarize the mechanism of action of baicalin in this article, including its anti-inflammatory and antioxidant effects; inhibition of endothelial cell apoptosis; modulation of innate immunity; suppression of VSMC proliferation, migration, and contraction; regulation of coagulation and fibrinolytic systems; inhibition of myocardial hypertrophy; prevention of myocardial fibrosis; and anti-apoptotic effects on cardiomyocytes. Notably, baicalin has a favorable anticoagulant effect, inhibiting the formation of atherosclerotic plaques. However, this activity is unfavorable for patients with blood coagulation disorders, and the clinical use of baicalin should be carefully considered in light of the patient’s medical history. Overall, although baicalin has shown many advantages in experimental studies, its unspecified underlying mechanisms still need to be explored in many studies. Current progress in the pharmacological studies of baicalin has led to the need to target specific organs to improve its utilization. A comprehensive study of baicalin and a targeted analysis of key issues are necessary.

## Figures and Tables

**Table:** 

**CHM**	chinese herbal medicine
**AS**	atherosclerosis
**MIRI**	myocardial ischemia‒reperfusion injury
**BG1**	baicalein-7-O-α-D-glucuronidyl-(1→4′)-O-α-D-glucopyranoside
**BG2**	baicalein-7-O-α-D-glucuronidyl-(1→4′)-O-α-D-maltoside
**BA-CMC**	carboxymethyl cellulose suspension containing BA
**BA-LPs**	BA-loaded liposomes
**SLNs**	solid lipid nanoparticles
**OX26-PEG-CSLN**	PEGylated cationic solid lipid nanoparticles loaded with baicalin
**SOL**	solution
**BN-PEG-NLCs**	baicalin-loaded PEGylated nanostructured lipid carriers
**SPIP**	situ single-pass intestine perfusion
**CDs**	cyclodextrins
**GUS**	β-glucuronidase
**GH**	glycoside hydrolase
**UGT**	UDP glucuronosyltransferase
**MRP2**	multidrug resistance protein 2
**MKK3**	mitogen-activated protein kinase kinase 3
**MKK6**	mitogen-activated protein kinase kinase 6
**NLRP3**	nucleotide-binding oligomerization domain-like receptor protein 3
**OS**	oxidative stress
**ROS**	reactive oxygen species
**MDA**	malondialdehyde
**SOD**	superoxide dismutase
**GSH-px**	glutathione peroxidase
**HO-1**	heme oxygenase-1
**GST**	glutathione S-transferase
**CAT**	catalase
**NOX**	NADPH oxidase
**GPX**	glutathione peroxidase
**PKCδ**	protein kinase C-delta
**RIP**	receptor-interacting protein
**RIP1**	receptor-interacting protein 1
**RIP3**	receptor-interacting protein 3
**MLKL**	mixed lineage kinase domain-like protein
**ERK**	extracellular regulated protein kinases
**PAR-1**	protease activated receptor-1
**ABCA1**	ATP binding cassette subfamily A member 1
**ABCG1**	ATP binding cassette subfamily G member 1
**SR-BI**	scavenger receptor class B type I
**PPAR-γ**	peroxisome proliferator-activated receptor-γ
**LXRα**	liver X receptor α
**IRF5**	interferon regulatory factor 5
**IRF4**	interferon regulatory factor 4
**MERTK**	myeloid-epithelial-reproductive tyrosine kinase
**PTX3**	pentraxins 3
**JAK2**	janus kinase 2
**STAT3**	signal transducer and activator of transcription 3
**SM22α**	smooth muscle 22α protein
**MEK**	mitogen-activated proteinkinase kinase 1
**ACE**	angiotensin I-converting enzyme
**Ang I**	angiotensin I
**Ang II**	angiotensin II
**PAI-1**	plasminogen activator inhibitor-1
**PT**	prothrombin time
**aPTT**	activated partial thromboplastin time
**TSP-1**	thrombospondin-1
**ICAM**	the adhesion molecules intercellular adhesion molecule
**VCAM**	vascular cell adhesion molecule
**LPS**	lipopolysaccharide
**ERKs**	Extracellular signal-regulated kinases
**JNKs**	c-Jun N-terminal kinases
**NLRP3**	nucleotide-binding oligomerization domain-like receptor protein 3
**PRRs**	pattern recognition receptors
**CCs**	cholesterol crystals
**LDL**	low-density lipoprotein
**GSDM**	gasdermin
**GPCR124**	G protein-coupled receptor 124
**TXNIP**	thioredoxin interacting protein
**ECs**	endothelial cells
**BA**	baicalin
**TLR4**	toll-like receptor 4
**MyD88**	myeloid differentiation primary response protein 88
**TNF-α**	tumor necrosis factor-α
**MAPK**	mitogen-activated protein kinase
**MAPKK**	MAPK kinase
**MAPKKK**	MAPK kinase kinase
**JUK**	c-Jun N-terminal kinase
**HAECs**	Human arterial endothelial cells
**H/R**	hypoxia/reoxygenation
**CMECs**	cardiac microvascular endothelial cells
**SR-A**	the type A scavenger receptor
**ApoA1**	apolipoprotein A1
**RCT**	reverse cholesterol transport
**TC**	total cholesterol
**SR**	The scavenger receptor
**SR-BI**	scavenger receptor class B type I
**VSMCs**	vascular smooth muscle cells
**α-SMA**	smooth muscle α-actinin
**SM22α**	smooth muscle 22α protein
**OPN**	osteopontin
**PDGF**	platelet-derived growth factor
**PDGFR**	the PDGF receptor
**RAS**	The renin–angiotensin system
**AT1R**	angiotensin II type 1 receptor
**MLCK**	myosin light chain kinase
**MLC**	myosin light chain
**HIF-1α**	Hypoxia-inducible factor 1
**JNK**	c-Jun N-terminal kinase
**BNIP3**	BCL2 interacting protein 3
**Drp1**	dynamin-related protein 1
**ALDH2**	aldehyde dehydrogenase 2
**AMP**	adenosine 5-monophosphate
**AMPK**	activated protein kinase
**mTOR**	mammalian target of rapamycin
**Bcl-2**	B-cell lymphoma-2
**Bax**	BCL2 associated X
**HMGB1**	high mobility group protein
**RAGE**	receptor for advanced glycation end products
**PPARα**	proliferator-activated receptor α
**TGF-β**	transforming growth factor-β
**Smad**	*Drosophila* mothers against decapentaplegic protein
**MMP-2**	metalloproteinases-2
**MMP-9**	metalloproteinases-9
**MMPs**	matrix metalloproteinases
**CTGF**	connective tissue growth factor
**I/R**	ischemia/reperfusion
**ALDH2**	aldehyde dehydrogenase 2
**FAO**	fatty acid oxidation
**MF**	myocardial fibrosis
**ECM**	extracardiac matrix
**FN**	fibronectin
